# Developing principles for sharing information about potential trial intervention benefits and harms with patients: report of a modified Delphi survey

**DOI:** 10.1186/s13063-022-06780-1

**Published:** 2022-10-08

**Authors:** Martina Svobodova, Nina Jacob, Kerry Hood, Katie Gillies, Rachel Hale, Jennifer Bostock, Peter Bower, Adrian Edwards, Penelope Farthing, Sarah Rawlinson, Shaun Treweek, Jeremy Howick

**Affiliations:** 1 Centre for Trials Research (CTR), College of Biomedical and Life Sciences, Cardiff University, 6th Floor, Neuadd Meirionnydd, Heath Park, Cardiff, CF14 4YS UK; 2 Health Services Research Unit, University of Aberdeen, Aberdeen, UK; 3 School of Psychology, Cardiff University, Cardiff, UK; 4 Division of Population Health, Health Services Research & Primary Care, University of Manchester, Manchester, UK; 5 Division of Population Medicine, School of Medicine, Cardiff University, Cardiff, UK; 6 Stoneygate Centre for Excellence in Empathic Healthcare, University of Leicester, Leicester, UK

**Keywords:** Clinical trials, Risk communication, Participant information leaflet, Nocebo effect, Medical risk factors

## Abstract

**Background:**

The way information about potential harms of trial intervention is shared within participant information leaflets (PILs) varies widely and can cause subjective ‘nocebo’ harms. This study aimed to develop principles to improve the composition of information about potential trial intervention benefits and harms within PILs so that variability and avoidable harms are reduced.

**Methods:**

We conducted a two-round modified online Delphi survey, followed by a consensus meeting. For the first round of the survey, 27 statements were developed based on previous research and relevant guidance from the UK, the USA and the World Health Organization. Participants included members from each of the following stakeholder groups: patient and public representatives, research ethics committee members, industry representatives, medico-legal experts, psychologists and trial managers. Each participant was asked to rate their degree of agreement or disagreement with each statement on a 9-point Likert scale. In the second round, participants were invited to reappraise their ratings after reviewing the results of the first round. Finally, two members from each stakeholder group participated in a meeting to confirm those statements for which there was agreement.

**Results:**

Two hundred and fifty participants completed round 1, and 201 participants completed round 2. In round 1, consensus was reached for 16 statements. In round 2, consensus was reached for an additional three statements. The consensus meeting confirmed the survey results and consolidated the statements. This process resulted in seven principles: (1) all potential harms of a given intervention should be listed, (2) all potential harms should be separated into serious and less serious, (3) it must be made explicit that not all potential harms are known, (4) all potential benefits should be listed, (5) all potential benefits and harms need to be compared with what would happen if the participant did not take part in the trial, (6) suitable visual representations should be added where appropriate and (7) information regarding potential benefits and harms should not be presented apart by one or more pages.

**Conclusions:**

Our modified Delphi process successfully generated seven principles that can and should be used to guide how information is conveyed to patients in information leaflets regarding potential trial benefits and harms.

**Supplementary Information:**

The online version contains supplementary material available at 10.1186/s13063-022-06780-1.

## Background

The potential benefits and risks of trial interventions are not communicated to patients in a consistent way. In a recent analysis of 33 participant information leaflets (PILs) used in trials conducted by the National Institute of Health Research (NIHR) in the UK [1], the way information about potential harms was communicated was found to be inconsistent. Most of the leaflets contained more information about harms than potential benefits, and some did not mention potential benefits at all. Failure to balance information about potential harms and benefits could harm trial participants. In a systematic review of over 250,000 trial participants who were given placebos, half of them reported having at least one negative side effect [2]. One in 20 of the participants who took a placebo refrained from participating further in the trial due to such side effects. This could be due to misattribution (whereby a symptom that would have arisen whether or not the patient participated in the trial is attributed to the trial intervention), negative expectations or ‘nocebo effects’. Nocebo effects are produced by negative expectations [3]. A trial participant might have been warned about a possible side effect in a way that caused them to expect, and consequently experience, this side effect. Nocebo effects are most commonly pain-related but can also include nausea, anxiety and other symptoms [2, 3].

The way information about potential trial treatment harms is communicated also poses an under-recognised ethical issue. The requirement of autonomy demands that trial participants be informed about all potential harms. However, if the way information about potential harms is conveyed causes harm, the ethical responsibility of non-maleficence may be violated. As far as we are aware, the ethical debate related to how information about potential trial treatment harms should be shared focuses on autonomy and neglects non-maleficence [3].

At present, no guidance is available that explains how to present information about potential trial benefits and harms in a way that respects the need to share information (respecting autonomy) and that is balanced (less likely to induce nocebo effects). Therefore, every principal investigator must negotiate their own method for sharing information about trial benefits and harms in a balanced way. This leads to the heterogeneity and increased risk of nocebo effects noted above. Ultimately, these biases in primary studies may lead to biases in meta-analyses and distort evidence of intervention effects that may affect judgements about effectiveness, cost-effectiveness and efficiency.

This study aimed to develop consensus-based principles to guide how information about trial intervention harms and benefits should be shared with patients so that unnecessary variation and harm is minimized.

## Methods

### Study design

Following a published protocol [4], we used a modified Delphi survey and the Guidance on Conducting and Reporting Delphi Studies (CREDES) to report this study [5] (see Additional file 1). The Delphi method is recommended for developing guidance, an expert meeting at the end can be superior for maximising cooperation between interdisciplinary researchers [6] and has been used successfully in similar areas [3, 7].

### Development of the list of statements for the Delphi survey

We generated a list of potential information about benefits and harms from three sources that background research suggested to be important:

1. Principles and examples from our review of UK PILs [1]
2. Extracted principles and examples from a random sample of Drug Facts Boxes [8]
3. Statements from official guidance about presenting trial benefits and harms in PILs from within the UK (e.g. Health Research Authority (HRA) [9]) and internationally (e.g. European Medicines Agency (EMA) [10]; World Health Organization (WHO) [11]; the United States Food and Drug Administration (USFDA) [12])

The long list derived from these sources was deduplicated and piloted for face validity by our co-applicant group and patient and public representatives.

### Sample size

There is currently no standard method for determining sample size calculations for Delphi studies [13]. The criteria for selecting experts are most prominently based on their representing a particular profession or stakeholder group and are not derived statistically [5]. While five to ten people per expert group are considered adequate for content validation, we aimed to sample 20 people per stakeholder group (100 in total). This number was based on a conservative estimate of a 50% dropout rate between Delphi rounds and a study by Harman et al. that used a lower limit of ten for each stakeholder group [14]. Furthermore, other studies suggest a minimum number of panel members ranging from 10 to 20 panel members per area of expertise [15, 16].

To maximise responses across stakeholder groups and to achieve diverse representation, we monitored the survey responses and sent additional reminders to groups that had fewer responses. The total number of respondents per stakeholder group was reviewed between rounds.

### Participant identification

A group of stakeholders was identified from the contact lists, networks of co-applicants and patient and public representatives. The survey was only available in English due to the time limitations of the study. Stakeholders included representatives from each of the five following sources:

1. *Public and patient representatives*: Our patient and public representative (Jennifer Bostock) and advisory board member (Jono Broad) helped us identify these representatives from their networks, including Pain UK and People in Research, NIHR, the James Lind Alliance, the NIHR Centre for Engagement and Dissemination and health literacy groups [17].
2. *Research ethics committee members and other approval staff*: The principal investigator and co-applicants have contacts at the HRA who put the study team in touch with interested ethics committee members and chairs.
3. *Industry (including medico-legal experts)*: Experts were identified with help from our industry partners (including the Association of British Pharmaceutical Industry) and with input from the advisory group (which includes medico-legal experts).
4. *Applied researchers, psychologists and risk communicators*: These were identified by the principal investigator and co-applicants, who have extensive networks of psychologists with relevant expertise. The study team targeted psychologists with a range of relevant expertise, especially in risk communication and behavioural science.
5. *Research nurses, clinical trial managers and trialists*: Researchers were identified via the UK Clinical Research Collaboration (UKCRC), the Registered Centre for Trials Research at Cardiff University and the UK Trial Managers’ Network (UKTMN).

Because of the different medico-legal frameworks in different countries, we restricted our stakeholders to those based in the UK.

### Delphi survey: design

Survey data were collected using Qualtrics [18]. Participants were invited to participate by email and asked to complete the online Delphi questionnaire through a weblink embedded in the email. The process was conducted anonymously to reduce the risk of any single respondent’s responses dominating the process or conclusions. All data were handled in accordance with UK data protection regulations. No demographic data were collected to preserve anonymity.

Following the methods used in a related study [7], our Delphi survey was introduced with a brief overview of the aims, how the collected information would be used and stored and how the findings would be made available to participants. Electronic consent was requested at the start of the survey. Participants who did not consent were not included in the study, and their data was not recorded. In round 2, reminder emails were sent to non-responders.

Analyses were conducted on fully anonymised Qualtrics survey data. Descriptive statistics were used to summarise the results from each round. For each item, the distribution of scores was summarised by stakeholder groups alongside the total number of participants who scored the item. Participants were instructed to rate each item independently even if items appeared similar.

### Delphi survey: conduct

**Fig. 1:**
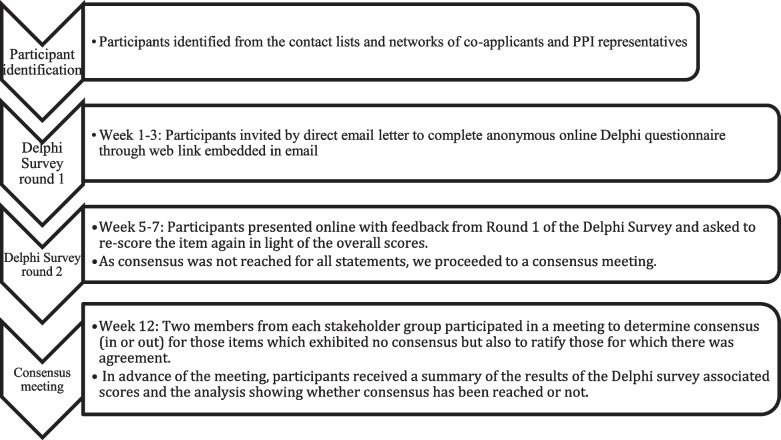
Participant flow diagram

The participant flow diagram (Fig. 1) outlines the progress of the Delphi survey participants through each stage. Rounds 1 and 2 of the Delphi survey were presented in an online format (see Additional file 2). Each participant’s email was automatically saved with their survey data. This allowed for the identification of individuals as they progressed through the Delphi process Responses were tracked while in progress, and reminders and thank you messages were sent out. Each respondent was asked to identify their stakeholder group (patient and advocates, research ethics committee members, etc.).

#### Round 1

In round 1, participants were presented with 27 statements across four sections. The first section included four hypothetical scenarios that described (i) a study participant being given excessive information about possible harms, (ii) not enough information about possible harms, (iii) a comparison of intervention with what would occur if a participant took nothing, and (iv) one scenario about positive framing. The second and third sections both related to describing the potential harms and benefits of a clinical trial. The fourth section included statements concerning the order and layout of benefits and harms in the participant leaflet. Participants were asked to rate their agreement or disagreement with the statements using a scale from one to nine, where one corresponds to ‘strongly agree’ and nine corresponds to ‘strongly disagree’. Participants were also given the opportunity to share free-text comments where they could provide reasons for their answers and propose further items for inclusion in round 2. Round 1 terminated when at least 20 participants across each of the stakeholder groups responded.

The research team then tabulated an overview of the total number of participants and each stakeholder group. For purposes of analysis, the scale was divided into clusters representing agreement (scoring 1–3), indecision (scoring 4–6) and disagreement (scoring 7–9) (see Additional file 3 for the full round 1 results).

#### Round 2

All participants taking part in round 1 were invited to participate in round 2 even if they completed the round 1 survey only partially. To maintain respondents’ engagement, the interval between the two rounds was restricted to 2 weeks.

Participants invited to round 2 were provided with a tabulated summary of the results of round 1, which included a list of statements that had reached consensus and a summary of the free-text comments (see Additional file 4). Consequently, participants could reflect on the results of the group and change their minds. Only items that did not achieve consensus in round 1 were required to be rated in round 2. Participants were asked to re-score the remaining statements after considering the summary results from round 1, using the same 9-point scale (see Additional file 5 for the full round 2 results).

The total number of participants invited to participate in round 2 was recorded and compared to the total number of round 1 responders. Statements that experts did not agree on were retained for discussion in the consensus meeting. Table 2 lists the 16 statements where consensus was reached following round 2.

### Definition and attainment of consensus

We defined consensus as follows:

- *Consensus in*: agreement of ≥ 70% of respondents that a principle should be followed when describing information about potential benefits and harms
- *Consensus out*: agreement of ≥ 70% of stakeholders that a principle should not be followed when describing information about potential benefits and harms
- *No consensus*: anything else

The cutoffs reflect recommended quality indicators for a Delphi study [19]. Items about which there was no consensus following round 2 were discussed in the in-person meeting, with the aim of either achieving consensus or agreeing on how to consider the lack of consensus in the eventual principles.

### Consensus meeting

For the final step of this modified Delphi method, we convened an online meeting with the co-applicants and two members from each stakeholder group. The meeting aimed to determine consensus (in or out) for those items which exhibited no consensus and to confirm those items for which there was agreement (see Additional file 6 for the statements discussed at the consensus meeting). All participants taking part in round 1 were contacted via email and invited to register their interest to participate in the consensus meeting. Out of the 49 participants that expressed their interest, we selected a group comprising 10 individuals balanced for gender and ethnicity. In advance of the meeting, participants received a brief summary of the results from each round of the Delphi survey and the analysis to reveal whether consensus had been reached or not.

The items that achieved consensus, together with accompanying free-text comments, were presented briefly, and participants were asked to voice any disagreement. The rest of the meeting focused on the items that did not reach consensus. Each statement was presented alongside its scores from individual stakeholder members and its corresponding free comments. Discussion was invited to clarify any points, and opportunities were given to consider whether each principle should be considered for inclusion. At the end of the meeting, the resulting final set of principles was presented to the group, and suggestions for simplifying and improving their expression were discussed.

## Results

### Respondent characteristics

Two hundred fifty stakeholders responded to the invitation email, gave informed consent and completed round 1 (see Table 1). A sufficient number of respondents (*n* ≥ 20 per stakeholder group) was achieved to progress to round 2. Two hundred one participants took part in round 2. The overall attrition rate between round 1 and round 2 was 19.6%, with the highest rate of 37.5% for industry stakeholders and the lowest rate of 8.3% for research ethics committee members and other approvals staff. Partial responses were included in the analysis for both rounds.

**Table 1:** Delphi survey participant characteristics

**Stakeholder group**	**Participants in round 1 (*n* = 250)**	**Participants in round 2 (*n* = 201)**
Public and patient representatives	57	46
Research ethics committee members and other approvals staff	36	33
Industry (including medico-legal experts)	24	15
Applied researchers, including psychologists and risk communicators	26	18
Research nurses, clinical trial managers and trialists	84	74
Others (including quality assurance managers, quality assurance auditors, clinical auditors, pharmacists, PhD students, sponsor representatives, research midwives and principal scientists)	23	15

The participants made free-text comments after both rounds (see Additional file 7 for a summary of all free text comments). In round 1, participants noted the need to clearly describe all potential risks and benefits and their degree of certainty as well as the necessity to tailor the presentation of risks depending on the disease type (see Additional file 8 for a full list of comments from round 1). The free-text comments from round 2 mentioned the need to balance severity and frequency. They also reiterated the need to communicate in a way that is understandable (see Additional file 9 for a full list of comments from round 2). The statements where consensus was reached following rounds 1 and 2 are shown in Table 2.

**Table 2:** Statements where consensus was reached following rounds 1 and 2

**‘Consensus in’: over 70% of respondents agreed with the following statements**	
**Statement no.**	**Statement**
2	Potentially serious harms need to be emphasised, even if they are very rare
3	Potential benefits and harms of a clinical trial need to be compared with what happens if the participant does not take part in the trial
7	The most likely potential benefits should be described
8	Any likely benefits to the participant (including embryos, foetus, nursing infants) should be described
9	General potential benefits (such as ‘the medicine may help you and your cancer’) should be described
10	Concrete, specific potential benefits (such as ‘this medicine is designed to enable you to walk farther before becoming breathless’) should be described
15	The harms should be separated into serious (life-threatening, causing permanent damage) and less serious (like a mild headache that goes away quickly)
16	Not all potential harms are known, especially for new treatments that have not been studied extensively. Participants need to know that not all potential harms can be listed
17	Sometimes harms are discovered after the trial begins. As soon as they are discovered, participants need to be told about them
18	Risks to conceiving/fathering a child, pregnancy or breastfeeding should be emphasised
21	Potential trial harms should be described in such a way that they can be compared to what would happen if the participant did not take part in the trial
**‘Consensus out’: over 70% of respondents disagreed with the following statements**	
**Statement no.**	**Statement**
5	Benefits are never completely certain, so they should not be described
6	Potential benefits should be described more fully than potential harms
12	Participants should not be told about potential harms
14	Only the most common possible harms should be mentioned. This will focus the reader’s attention and minimise overload
24	Information about potential benefits or harms should be presented apart by one or more pages

### Consensus meeting

The participants in the consensus meeting approved the 16 statements (11 in and five out) from the Delphi survey (see Additional file 10 for a full description of the meeting). They were given the option of objecting to the statements, but no objections were raised. Regarding the statements about which there was no consensus, the group discussed at length the suitability of using visual representations to assist in describing potential risks and harms. In the survey, consensus for the statement regarding the inclusion of visual representation was not reached (32.7% agreed, 50.4% undecided and 16.8% disagreed in round 1; 29.22% agreed, 54.49% undecided and 16.3% disagreed in round 2). Yet, because of the overwhelming support and the fact that visual representations were not mandated (only recommended 'where appropriate'), the group voted for and approved the inclusion of a modified version of statement 20: *Suitable visual representations are recommended where appropriate to describe potential intervention benefits and harms, such as the happy and sad faces.*

The consensus meeting also assisted in resolving some differences between responses across the stakeholder groups in previous rounds and in harmonising some of the statements in several areas:

1. The statement, *Only the most important potential benefits should be described. If too many are included the reader might become confused. A complete list can be contained in an appendix or online*, went from no consensus in the first round to consensus amongst some stakeholder groups (ethics committee members and industry) but not overall. After discussion, the consensus meeting attendees agreed that this statement was redundant, and that potential benefits should always be listed.
2. The statement, *Information about potential benefits and harms should be mentioned in more than one place in the leaflet*, reached consensus amongst applied researchers and clinical trial professionals but not overall. The consensus meeting attendees agreed that PILs should be clear and concise and that anything that makes them longer and more complicated than necessary should be avoided. Repetition of benefits and harms in more than one place would not add any value, so the group agreed to leave this statement out.
3. The statement, *A complete (detailed) description of the potential harms (and the likelihood of each harm) should be provided in a table in an appendix*, reached consensus amongst the research ethics committee members and ‘near’ consensus amongst the public, patients and their advocates and clinical trial professions groups. The consensus group meeting attendees recognised that some potential trial participants might want condensed information on potential harms while others will prefer more detailed information that an appendix could provide. In the end, the meeting attendees agreed to leave the statement out.
4. The statement, *Drug fact boxes... divide harms into serious and non-serious. This way of presenting harms is helpful*, reached consensus amongst the public, patients and their advocates and applied researchers. The consensus group, while acknowledging that drug fact boxes are a useful clinical tool, found that there was a level of uncertainty regarding the generalizability of the tool to non-drug treatments and trials. Consequently, a consensus was not reached.

Once full consensus was reached on the statements, the participants spent the remainder of the in-person meeting discussing aesthetic modifications to the final set of principles, including the possibility of grouping similar principles together to remove any repetition. The final principles, together with the survey statements that they are based on, are shown in Table 3 (See Additional file 11 for further information).

**Table 3:** Set of core principles as approved by the consensus meeting

**Principle no**	**Based on statements**	**Description**
1	2, 18	All potential harms of the intervention should be listed. This includes the following: - Common as well as rare potential harms - Indirect potential harms (for example, to conceiving a child, pregnancy or breastfeeding)
2	15	The harms should be separated into serious (life-threatening, causing permanent damage) and less serious (like a mild headache that goes away quickly)
3	17	The fact that not all potential harms are known needs to be explicit. Also, sometimes, harms are discovered after the trial begins. As soon as they are discovered, participants need to be told about them
4	7, 8, 9, 10	All potential benefits of the intervention should be listed. This includes the following: - General potential benefits (such as ‘the medicine may help you and your cancer’) should be described - Concrete, specific potential benefits (such as ‘this medicine is designed to enable you to walk farther before becoming breathless’) should be described - Likely benefits to the participant (including embryos, foetus, nursing infants) should be described
5	3	Potential benefits and harms of a clinical trial need to be compared with what happens if the participant does not take part in the trial
6	20	Suitable visual representations are recommended where appropriate to describe potential intervention benefits and harms, such as pictograms of faces
7	Negated 24	Information about potential benefits and harms should not be presented apart by one or more pages

## Discussion

### Summary of findings

We were able to identify seven principles to guide how information is shared regarding potential trial intervention benefits and harms. The principles are based on consensus exercises involving a wide range of stakeholders.

### Context of other literature

Guidance for sharing information about potential trial benefits and harms within PILs is under-researched. An exception may be ‘Drug Facts Boxes’, which were developed to improve patient understanding of drug benefits and harms [8]. However, Drug Facts Boxes apply exclusively to the pharmaceutical setting and are not directly applicable to the UK regulatory research context. Relatedly, one study found that pharmacists changed the treatments they provided to patients depending on how risks were communicated to them [20]. A recent systematic review also found that there was not yet a clear, optimal method for communicating risks to patients within trials [21]. Our study thus represents an important step forward on existing literature by providing clear consensus-based principles that can guide the description of potential trial intervention benefits and harms to participants.

### Limitations

Despite the large sample for this type of study (more than double what we planned for), the stakeholders may not have been representative of all relevant parties. We believe that the high retention rates and the clear consensus on most items mitigate the impact of this potential problem.

### Implications for research

The principles developed by our exercise can now be used to design the relevant sections of PILs. PILs developed according to these principles should be rigorously compared with other PILs to check whether they reduce nocebo effects or improve recruitment and retention rates. This could be straightforwardly achieved by using ‘studies with a trial’ (‘SWATs’).

The seven core principles described in this study can also be used to inform future HRA guidance on sharing information about potential trial treatment benefits and harms. Importantly, the principles revealed by the rigorous process of this study are broadly in line with current HRA guidance; however, there are some important differences. For example, whereas current relevant HRA guidance is brief (which could be a cause of the variability) [1], our guidance is more extensive. Another important difference is that whereas current HRA guidance states that it is not usually possible to specify potential benefits, our stakeholders were clear that potential benefits (which are not certain benefits) should be listed. We also note that there is no difference *in principle* between an effect that is harmful and an effect that is beneficial [22]. In fact, effects that are harmful to some could be beneficial to others. For example, a relatively common side effect of SSRIs is sexual dysfunction [23]. This is a negative effect of SSRIs for many people, but the very same phenomenon is a positive effect for people with premature ejaculation. In other cases, the same drug can cause one effect in some people and a (paradoxical) opposite effect in others [24]. For example, amphetamines are stimulants for most people yet cause drowsiness in some [25]. Therefore, if potential harms can be mentioned, there is no logical justification for omitting potential benefits.

In addition, practical guidance on how to implement the principles could be produced. This would serve to reduce variability in the way trial benefits and harms are described. Clear guidance could reduce the time spent by those who design PILs and ethics committees who review them. To be implemented, additional research would need to be done about the differences between drug and non-drug trials. Potential harms of drugs are usually listed comprehensively (the study protocol, reference safety information such as the investigator’s brochure or summary of product characteristics), but for non-drug trials, additional research is often required to derive a complete list of potential harms.

Our study also contributes to recent ethical research, which has found that research ethics committees are overly focused on one interpretation of the principle of autonomy, leading to an over-emphasis on describing potential harms [3]. The principles described here illustrate that respecting autonomy also demands that information about potential benefits is not withheld. Consequently, research ethics committees should be made aware of these principles.

These principles should be adapted for at least two related areas: clinical practice and instruction about how to verbally communicate information about potential benefits and harms. In clinical practice, healthcare practitioners could use a version of these principles to describe potential benefits and harms to their patients. Relatedly, a training package that explains how these principles should be explained verbally could be developed for research nurses and others who take informed consent. Our principles could also be tested in different countries that do not share the same medico-legal context as the UK.

## Conclusions

Clear consensus about seven principles to guide the way potential benefits and harms or trial treatments are described within patient information leaflets was reached from a wide range of stakeholders. These principles can now be implemented to design and evaluate patient information leaflets to reduce variability in the way in which information about potential benefits and harms of trial treatments are described.

## Supplementary Information

**Additional file 1.** CREDES Checklist.

**Additional file 2.** Delphi survey instruction.

**Additional file 3.** Full round 1 results.

**Additional file 4.** Summary round 1 results.

**Additional file 5.** Full round 2 results.

**Additional file 6.** Consensus meeting statements.

**Additional file 7.** Round 1 & 2 free-text comments.

**Additional file 8.** Round 1 free-text comments.

**Additional file 9.** Round 2 free-text comments.

**Additional file 10.** Consensus meeting description.

**Additional file 11.** Final set of principle statements.

## Data Availability

All data generated or analysed during this study are included in this published article and its supplementary information files.

## Abbreviations

PIL: Patient information leaflet
NIHR: National Institute of Health Research
HRA: Health Research Authority
EMA: European Medicines Agency
WHO: World Health Organization
USFDA: United States Food and Drug Administration
UKCRC: UK Clinical Research Collaboration
UKTMN: UK Trial Managers’ Network
