# Supplementary material for: Developing principles for sharing information about potential trial intervention benefits and harms with patients: report of a modified Delphi survey
Source: Trials. 2022 Oct 8;23:863. doi: 10.1186/s13063-022-06780-1 (PMC9548137; doi:10.1186/s13063-022-06780-1)
Supplement: Supplementary file 3 — Additional file 3. Full round 1 results. [file 13063_2022_6780_MOESM3_ESM.pdf]

File name: Additional file 3

File format: .doc

Title: Full Round 1 Results Table

Description: Full results from round 1 of the Delphi survey

**Question No.**	**Statement**	**Score 1-3**	**% of N**	**Score 4-6**	**% of N**	**Score 7-9**	**% of N**	**Decision**
1	Potential harms that are not very serious do not need to be emphasized.	**53**	**23.14**	**62**	**27.07**	**114**	**49.78**	**No consensus**
	Public, Patient and their advocate	18	33.69	11	20.76	24	45.27	No consensus
	Ethics committee member etc.	5	13.89	11	30.56	20	55.56	No consensus
	Industry (inc. medico-legal expert)	2	9.52	6	28.57	14	61.91	No consensus
	Applied researcher	5	20.84	7	29.17	12	50	No consensus
	Clinical trial professionals	21	26.92	20	25.64	37	47.44	No consensus
	Other	2	10	6	30	12	60	No consensus
2	Potentially serious harms need to be emphasized, even if they are very rare.	**188**	**81.73**	**35**	**15.21**	**5**	**3.03**	**Consensus**
	Public, Patient and their advocate	45	84.91	4	13.21	1	1.89	Consensus
	Ethics committee member etc.	29	80.55	6	16.67	1	2.78	Consensus
	Industry (inc. medico-legal expert)	16	76.19	2	9.52	3	14.28	Consensus
	Applied researcher	19	79.16	4	16.67	1	4.17	Consensus
	Clinical trial professionals	63	79.75	14	17.72	2	2.54	Consensus
	Other	18	90	2	10	0	0	Consensus
3	Potential benefits and harms of a clinical trial need to be compared with what happens if the participant does not take part in the trial.	**184**	**80.35**	**34**	**14.85**	**11**	**3.93**	**Consensus**
	Public, Patient and their advocate	45	84.91	7	13.21	1	1.89	Consensus

	Ethics committee member etc.	31	86.11	1	2.78	4	11.12	Consensus
	Industry (inc. medico-legal expert)	19	90.48	2	9.52	0	0	Consensus
	Applied researcher	19	79.16	4	16.67	1	4.17	Consensus
	Clinical trial professionals	59	74.23	15	19.23	4	5.12	Consensus
	Other	15	75	4	20	1	5	Consensus
4	It is okay to use ‘positive framing’ when describing how severe harms can be.	**90**	**39.3**	**72**	**31.44**	**67**	**29.26**	**No consensus**
	Public, Patient and their advocate	24	45.28	18	33.97	11	20.76	No consensus
	Ethics committee member etc.	8	22.22	13	36.11	15	41.68	No consensus
	Industry (inc. medico-legal expert)	8	38.1	4	19.05	9	24.85	No consensus
	Applied researcher	10	41.67	7	29.16	7	29.16	No consensus
	Clinical trial professionals	31	40.01	28	35.9	19	24.35	No consensus
	Other	9	45	3	15	8	40	No consensus
5	Benefits are never completely certain, so they should not be described.	**8**	**3.49**	**45**	**19.65**	**176**	**76.85**	**Consensus**
	Public, Patient and their advocate	4	7.54	12	22.64	37	69.81	No consensus
	Ethics committee member etc.	2	5.71	7	20	26	74.28	Consensus
	Industry (inc. medico-legal expert)	0	0	4	19.04	17	80.96	Consensus
	Applied researcher	1	4.17	4	16.67	19	79.17	Consensus
	Clinical trial professionals	2	2.60	13	16.89	62	80.51	Consensus
	Other	0	0	4	20	16	80	Consensus
6	Potential benefits should be described more fully than potential harms.	**15**	**6.58**	**54**	**23.69**	**159**	**69.74**	**No consensus**
	Public, Patient and their advocate	5	9.62	18	34.61	29	65.4	No consensus
	Ethics committee member etc.	1	2.86	5	14.29	29	82.86	Consensus

	Industry (inc. medico-legal expert)	0	0	1	4.76	20	96.24	Consensus
	Applied researcher	0	0	6	25	18	75	Consensus
	Clinical trial professionals	4	5.2	18	28.57	55	71.43	Consensus
	Other	2	10	7	35	11	55	No consensus
7	The most likely potential benefits should be described.	**188**	**82.1**	**34**	**14.84**	**7**	**3.06**	**Consensus**
	Public, Patient and their advocate	45	84.9	7	13.2	1	1.89	Consensus
	Ethics committee member etc.	28	80	7	20	0	0	Consensus
	Industry (inc. medico-legal expert)	18	85.71	2	9.52	1	4.76	Consensus
	Applied researcher	21	87.5	2	8.34	1	4.17	Consensus
	Clinical trial professionals	64	83.12	12	15.58	1	1.30	Consensus
	Other	11	55	7	35	2	10	No consensus
8	Any likely benefits to the participant (including embryos, foetus, nursing infants) should be described.	**181**	**79.13**	**46**	**20.07**	**2**	**0.87**	**Consensus**
	Public, Patient and their advocate	42	79.25	11	20.75	0	0	Consensus
	Ethics committee member etc.	28	80	7	20	0	0	Consensus
	Industry (inc. medico-legal expert)	15	71.44	5	23.8	1	4.76	Consensus
	Applied researcher	20	83.34	4	16.67	0	0	Consensus
	Clinical trial professionals	61	79.23	14	18.18	2	2.60	Consensus
	Other	13	65	7	35	0	0	No consensus
9	General potential benefits (such as ‘the medicine may help you and your cancer’) should be described.	**146**	**64.04**	**63**	**27.63**	**19**	**8.33**	**No consensus**
	Public, Patient and their advocate	33	63.46	15	28.85	4	7.69	No consensus
	Ethics committee member etc.	21	60	12	34.29	2	5.71	No consensus
	Industry (inc. medico-legal expert)	14	76.18	3	14.28	2	9.52	Consensus
	Applied researcher	12	49.99	9	37.5	3	12.5	No consensus
	Clinical trial professionals	51	66.23	20	25.96	6	7.79	No consensus
	Other	11	55	6	30	3	15	No consensus

10	Concrete, specific potential benefits (such as ‘this medicine is designed to enable you to walk farther before becoming breathless’) should be described.	**194**	**70.18**	**24**	**10.53**	**10**	**4.38**	**Consensus**
	Public, Patient and their advocate	47	90.39	3	5.77	2	3.85	Consensus
	Ethics committee member etc.	26	74.28	7	20	2	5.71	Consensus
	Industry (inc. medico-legal expert)	13	61.91	6	28.57	2	9.52	No consensus
	Applied researcher	24	95.83	1	4.17	0	0	Consensus
	Clinical trial professionals	67	87.02	6	7.79	4	5.20	Consensus
	Other	18	90	2	10	0	0	Consensus
11	Only the most important potential benefits should be described. If too many are included the reader might become confused. A complete list can be contained in an appendix or online.	**113**	**33.63**	**77**	**33.63**	**39**	**17.03**	**No consensus**
	Public, Patient and their advocate	21	39.62	15	28.3	17	32.08	No consensus
	Ethics committee member etc.	16	45.71	14	40	5	14.28	No consensus
	Industry (inc. medico-legal expert)	13	61.91	3	14.28	5	23.81	No consensus
	Applied researcher	12	50.01	10	41.67	2	8.34	No consensus
	Clinical trial professionals	39	50.65	28	36.36	10	12.99	No consensus
	Other	12	60	6	30	2	10	No consensus
12	Participants should not be told about potential harms.	**13**	**5.72**	**3**	**1.32**	**211**	**92.95**	**Consensus**
	Public, Patient and their advocate	4	7.84	1	1.96	46	90.19	Consensus
	Ethics committee member etc.	2	5.88	0	0	32	94.12	Consensus
	Industry (inc. medico-legal expert)	1	5	0	0	19	95	Consensus
	Applied researcher	0	0	0	0	24	100	Consensus
	Clinical trial professionals	5	6.58	0	0	71	93.43	Consensus
	Other	0	0	2	10	18	90	Consensus

13	Potential harms should be described more fully than potential trial benefits.	**42**	**18.42**	**74**	**32.45**	**112**	**49.12**	**No consensus**
	Public, Patient and their advocate	10	19.23	19	36.54	23	44.23	No consensus
	Ethics committee member etc.	10	29.41	7	20.58	17	49.99	No consensus
	Industry (inc. medico-legal expert)	6	30	5	25	9	45	No consensus
	Applied researcher	3	12.5	10	41.66	11	45.84	No consensus
	Clinical trial professionals	9	11.85	27	35.52	40	52.63	No consensus
	Other	4	20	7	35	9	45	No consensus
14	Only the most common possible harms should be mentioned. This will focus the reader's attention and minimize overload.	**35**	**15.32**	**80**	**35.09**	**134**	**58.77**	**No consensus**
	Public, Patient and their advocate	9	17.31	12	23.09	31	59.62	No consensus
	Ethics committee member etc.	5	14.7	5	14.7	24	70.59	Consensus
	Industry (inc. medico-legal expert)	2	10	6	30	12	60	No consensus
	Applied researcher	2	8.33	3	12.5	19	79.17	Consensus
	Clinical trial professionals	13	17.11	26	34.22	37	48.68	No consensus
	Other	2	10	9	45	9	45	No consensus
15	The harms should be separated into serious (life threatening, causing permanent damage) and less serious (like a mild headache that goes away quickly).	**195**	**85.53**	**25**	**10.96**	**8**	**3.51**	**Consensus**
	Public, Patient and their advocate	39	80.78	7	13.46	3	5.76	Consensus
	Ethics committee member etc.	32	94.12	2	5.88	0	0	Consensus
	Industry (inc. medico-legal expert)	18	90	1	5	1	5	Consensus
	Applied researcher	22	91.66	1	4.17	1	4.17	Consensus
	Clinical trial professionals	64	84.21	10	13.16	2	2.64	Consensus
	Other	15	75	4	20	1	5	Consensus
16	Not all potential harms are known, especially for new treatments that have not been studied extensively. Participants need	**207**	**90.79**	**19**	**8.33**	**2**	**0.88**	**Consensus**

	to know that not all potential harms can be listed.							
	Public, Patient and their advocate	48	88.46	6	11.54	0	0	Consensus
	Ethics committee member etc.	32	94.12	2	5.88	0	0	Consensus
	Industry (inc. medico-legal expert)	20	100	0	0	0	0	Consensus
	Applied researcher	23	95.83	1	4.17	0	0	Consensus
	Clinical trial professionals	67	88.16	9	11.84	0	0	Consensus
	Other	17	85	1	5	2	10	Consensus
17	Sometimes harms are discovered after the trial begins. As soon as they are discovered, participants need to be told about them.	**208**	**91.63**	**18**	**7.93**	**1**	**0.44**	**Consensus**
	Public, Patient and their advocate	48	94.11	3	5.88	0	0	Consensus
	Ethics committee member etc.	31	91.18	3	8.82	0	0	Consensus
	Industry (inc. medico-legal expert)	19	90	2	10	0	0	Consensus
	Applied researcher	22	91.66	1	4.17	1	4.17	Consensus
	Clinical trial professionals	71	93.43	5	6.58	0	0	Consensus
	Other	17	85	3	15	0	0	Consensus
18	Risks to conceiving/fathering a child, pregnancy, or breastfeeding should be emphasized.	**197**	**86.78**	**28**	**12.34**	**6**	**2.64**	**Consensus**
	Public, Patient and their advocate	42	82.35	7	15.68	1	1.96	Consensus
	Ethics committee member etc.	32	94.12	2	5.88	0	0	Consensus
	Industry (inc. medico-legal expert)	17	85	3	15	0	0	Consensus
	Applied researcher	19	79.17	5	20.83	0	0	Consensus
	Clinical trial professionals	69	90.8	6	7.89	1	1.32	Consensus
	Other	17	85	3	15	0	0	Consensus
19	It’s okay to use ‘positive framing’. That is, it is okay to say ‘this treatment is safe for 90% of the people who take it’ instead of ‘this	**45.37**	**103**	**74**	**32.6**	**50**	**9.69**	**No consensus**

	treatment causes side effects for 10% of the people who take it'.							
	Public, Patient and their advocate	29	56.87	11	21.56	11	21.56	No consensus
	Ethics committee member etc.	10	29.41	16	47.06	8	23.53	No consensus
	Industry (inc. medico-legal expert)	8	40	5	25	7	35	No consensus
	Applied researcher	13	54.16	7	29.17	4	16.67	No consensus
	Clinical trial professionals	37	48.68	25	32.89	14	18.42	No consensus
	Other	7	35	8	40	5	25	No consensus
20	Potential harms should be described in pictures as well as words.	**74**	**32.74**	**114**	**50.44**	**38**	**16.82**	**No consensus**
	Public, Patient and their advocate	25	49.01	21	41.17	4	9.8	No consensus
	Ethics committee member etc.	6	18.18	22	66.67	5	15.15	No consensus
	Industry (inc. medico-legal expert)	8	40	5	25	7	35	No consensus
	Applied researcher	8	33.34	10	41.67	6	25	No consensus
	Clinical trial professionals	15	19.73	48	63.17	13	17.11	No consensus
	Other	9	45	8	40	3	15	No consensus
21	Potential trial harms should be described in such a way that they can be compared to what would happen if participant did not take part in the trial.	**175**	**77.09**	**41**	**18.06**	**11**	**4.84**	**Consensus**
	Public, Patient and their advocate	44	86.28	6	11.76	1	1.96	Consensus
	Ethics committee member etc.	26	76.48	7	20.58	1	2.94	Consensus
	Industry (inc. medico-legal expert)	15	75	4	20	1	5	Consensus
	Applied researcher	21	87.5	2	8.33	1	4.17	Consensus
	Clinical trial professionals	50	65.79	19	25	7	9.21	No consensus
	Other	15	75	4	20	1	5	Consensus
22	Potential benefits should be described after harms.	**27**	**11.84**	**119**	**52.19**	**82**	**35.96**	**No consensus**
	Public, Patient and their advocate	8	15.96	18	35.29	25	49.01	No consensus
	Ethics committee member etc.	7	21.21	21	63.63	5	15.15	No consensus

	Industry (inc. medico-legal expert)	5	25	8	40	7	35	No consensus
	Applied researcher	1	4.17	11	45.83	12	50	No consensus
	Clinical trial professionals	4	5.41	48	64.87	22		No consensus
	Other	2	10	12	60	6	30	No consensus
23	Potential benefits and harms should be beside each other (for example in two columns).	**97**	**42.74**	**92**	**40.53**	**38**	**16.73**	**No consensus**
	Public, Patient and their advocate	31	62	11	22	8	16	No consensus
	Ethics committee member etc.	7	21.21	18	54.54	8	24.24	No consensus
	Industry (inc. medico-legal expert)	6	30	6	30	8	40	No consensus
	Applied researcher	12	49.99	9	37.49	3	12.5	No consensus
	Clinical trial professionals	28	37.84	38	51.35	8	10.81	No consensus
	Other	9	54	8	40	3	20	No consensus
24	Information about potential benefits or harms should be presented apart by one or more pages.	**12**	**5.31**	**81**	**35.83**	**133**	**58.85**	**No consensus**
	Public, Patient and their advocate	7	14	14	28	29	58	No consensus
	Ethics committee member etc.	0	0	15	45.45	18	54.54	No consensus
	Industry (inc. medico-legal expert)	1	5	7	35	12	60	No consensus
	Applied researcher	1	4.17	3	12.5	20	83.34	Consensus
	Clinical trial professionals	2	2.74	31	42.47	40	54.8	No consensus
	Other	0	0	8	40	12	60	No consensus
25	Information about potential benefits and harms should be mentioned in more than one place in the leaflet.	**24**	**10.61**	**91**	**40.27**	**111**	**49.11**	**No consensus**
	Public, Patient and their advocate	7	14	20	40	23	46	No consensus
	Ethics committee member etc.	3	9.09	19	57.57	11	33.33	No consensus
	Industry (inc. medico-legal expert)	0	0	8	40	12	60	No consensus
	Applied researcher	1	4.17	10	41.67	13	54.17	No consensus

	Clinical trial professionals	4	5.4	27	36.48	43	58.11	No consensus
	Other	6	31.58	6	31.58	7	36.84	No consensus
26	A complete (detailed) description of the potential harms (and the likelihood of each harm) should be provided in a table in an appendix.	**114**	**50.22**	**90**	**39.65**	**23**	**10.13**	**No consensus**
	Public, Patient and their advocate	30	60	19	38	1	2	No consensus
	Ethics committee member etc.	16	48.48	14	42.42	3	9.09	No consensus
	Industry (inc. medico-legal expert)	9	45	6	30	5	25	No consensus
	Applied researcher	16	66.67	5	20.84	3	12.5	No consensus
	Clinical trial professionals	27	36.49	48	51.35	9	12.16	No consensus
	Other	13	65	6	30	1	5	No consensus
27	Drug fact boxes (see below) divide harms into serious and non-serious. This way of presenting harms is helpful.	**124**	**55.11**	**55**	**24.45**	**46**	**20.45**	**No consensus**
	Public, Patient and their advocate	26	53.06	12	24.49	11	22.45	No consensus
	Ethics committee member etc.	18	54.54	7	21.21	8	24.24	No consensus
	Industry (inc. medico-legal expert)	10	50	6	30	4	20	No consensus
	Applied researcher	18	75	3	12.5	3	12.5	Consensus
	Clinical trial professionals	39	52.7	18	24.33	17	22.97	No consensus
	Other	9	47.36	7	36.84	3	15.79	No consensus
