# Supplementary material for: Developing principles for sharing information about potential trial intervention benefits and harms with patients: report of a modified Delphi survey
Source: Trials. 2022 Oct 8;23:863. doi: 10.1186/s13063-022-06780-1 (PMC9548137; doi:10.1186/s13063-022-06780-1)
Supplement: Supplementary file 4 — Additional file 4. Summary round 1 results. [file 13063_2022_6780_MOESM4_ESM.pdf]

File name: Additional file 4

File format: .doc

Title: Delphi Round 1 Results & summary of free text comments

Description: Results from round 1 of the Delphi survey where consensus has and has not been reached. Summary of free text comments from participants. These results were given to all participants ahead of completion of round 2 of the Delphi survey.

Statements where consensus has been reached following round 1	
**Question #**	**Statement**
**Over 70% of respondents agreed with the following statements**	
2.	Potentially serious harms need to be emphasized, even if they are very rare.
3.	Potential benefits and harms of a clinical trial need to be compared with what happens if the participant does not take part in the trial.
7.	The most likely potential benefits should be described.
8.	Any likely benefits to the participant (including embryos, foetus, nursing infants) should be described.
10.	Concrete, specific potential benefits (such as ‘this medicine is designed to enable you to walk farther before becoming breathless’) should be described.
15.	The harms should be separated into serious (life threatening, causing permanent damage) and less serious (like a mild headache that goes away quickly).
16.	Not all potential harms are known, especially for new treatments that have not been studied extensively. Participants need to know that not all potential harms can be listed.
17.	Sometimes harms are discovered after the trial begins. As soon as they are discovered, participants need to be told about them.
18.	Risks to conceiving/fathering a child, pregnancy, or breastfeeding should be emphasized.
21.	Potential trial harms should be described in such a way that they can be compared to what would happen if participant did not take part in the trial.
**Over 70% of respondents disagreed with the following statements**	
5.	Benefits are never completely certain, so they should not be described.
6.	Potential benefits should be described more fully than potential harms.
12.	Participants should not be told about potential harms.

Summary of free text comments

Participants said it is important to clearly describe risks in terms of both how often the side effects arose and how serious they were.
Leaflets should avoid using ‘you’ or ‘your child’ and use more generic ‘some participants’ or ‘some children’.
Comparison to known medications can be useful when describing potential harms. For example, saying that the probability of a clot following a vaccine is the same as the probability of a clot linked to smoking or pregnancy.
Uncertainty needs to be conveyed (we are usually not sure who will experience a harm).

**Statements where consensus has not been reached following round 1**				
	**Section One: Scenarios**			
**#**	**Scenario**	**Scenario description**	**Statement (to score using Likert Scale)**	**Round 1 score**

**1.**	**Giving a participant too much information about possible harms**	John was thinking about enrolling in a trial of a new drug to treat migraines. He read the participant information leaflet very carefully, and in several places the leaflet contained information about gastrointestinal side effects. He didn't know what this meant so he looked it up and figured out that it probably meant stomach aches and nausea. He didn't find much information about the benefits of the new drug, but his doctor said it was worth a try and why would his doctor say that if it wasn't going to help? John enrolled in the trial, and his migraine symptoms got a bit better, but every evening he suffered from stomach aches and withdrew from the trial.	**Statement 1.** Potential harms that are not very serious do not need to be emphasized.	23% of participants agreed (score 1-3), 27% of participants were undecided (score 4-6) and 50% participants disagreed (score 7-9)
**4.**	**Positive framing**	Two trials were recruiting people with psoriasis to test new drugs. Andrew had psoriasis and asked about information about both trials. In the information leaflet describing the first trial, the drug was described as having 'common side-effects, affecting 1 in 10 people'. In the second, the side-effects were described as 'uncommon: 90% of people will not be affected.' Andrew felt safer enrolling in the second trial. *Saying (1) 'this drug has a common side effect that affects 1 in 10 people' is* logically *the same thing as saying (2) 'this drug has an uncommon side effect: 90% of people who take it are not affected.' The second way of saying it is called 'positive framing'.*	**Statement 4**. It is okay to use 'positive framing' when describing how severe harms can be.	39% of participants agreed (score 1-3), 31% of participants were undecided (score 4-6) and 29% participants disagreed (score 7-9)

Section Two: Describing potential benefits of a clinical trial		
**#**	**Statement (to score using Likert Scale)**	**Round 1 score**
**9.**	General potential benefits (such as ‘the medicine may help you and your cancer’) should be described.	64% of participants agreed (score 1-3), 28% of participants were undecided (score 4-6) and 8% participants disagreed (score 7-9)
**11.**	Only the most important potential benefits should be described. If too many are included the reader might become confused. A complete list can be contained in an appendix or online.	49% of participants agreed (score 1-3), 33% of participants were undecided (score 4-6) and 17% participants disagreed (score 7-9)
**Section 3: Describing potential harms of a clinical trial**		
**13.**	Potential harms should be described more fully than potential trial benefits.	18% of participants agreed (score 1-3), 32% of participants were undecided (score 4-6) and 49% participants disagreed (score 7-9)
**14.**	Only the most common possible harms should be mentioned. This will focus the reader’s attention and minimize overload.	15% of participants agreed (score 1-3), 26% of participants were undecided (score 4-6) and 59% participants disagreed (score 7-9)
**19.**	It’s okay to use ‘positive framing’. That is, it is okay to say ‘this treatment is safe for 90% of the people who take it’ instead of ‘this treatment causes side effects for 10% of the people who take it’.	46% of participants agreed (score 1-3), 32% of participants were undecided (score 4-6) and 22% participants disagreed (score 7-9)
**Section Four: The ordering and placement of benefits and harms in the participant leaflet / layout**		
**20.**	Potential harms should be described in pictures as well as words.	33% of participants agreed (score 1-3), 50% of participants were undecided (score 4-6) and 17% participants disagreed (score 7-9)

**22.**	Potential benefits should be described after harms.	43% of participants agreed (score 1-3), 41% of participants were undecided (score 4-6) and 17% participants disagreed (score 7-9)
**23.**	Potential benefits and harms should be beside to each other (for example in two columns).	43% of participants agreed (score 1-3), 41% of participants were undecided (score 4-6) and 17% participants disagreed (score 7-9)
**24.**	Information about potential benefits or harms should be presented apart by one or more Pages.	5% of participants agreed (score 1-3), 36% of participants were undecided (score 4-6) and 59% participants disagreed (score 7-9)
**25.**	Information about potential benefits and harms should be mentioned in more than one place in the leaflet.	11% of participants agreed (score 1-3), 40% of participants were undecided (score 4-6) and 49% participants disagreed (score 7-9)
**26.**	A complete (detailed) description of the potential harms (and the likelihood of each harm) should be provided in a table in an appendix.	50% of participants agreed (score 1-3), 40% of participants were undecided (score 4-6) and 10% participants disagreed (score 7-9)
**27.**	Drug fact boxes (see below) divide harms into serious and non-serious. This way of presenting harms is helpful.	55% of participants agreed (score 1-3), 25% of participants were undecided (score 4-6) and 20% participants disagreed (score 7-9)

What difference did ABILIFY make?	Anti-depressant + ABILIFY (10 mg each day)	vs.	Anti-depressant + PLACEBO (No drug)
**How did ABILIFY help?**			
Depression scores improved by 3 points more than placebo (on a scale from 0 to 60).	9 points better	vs.	6 points better
11% more people had an important response and were no longer considered to have major depression	26%	vs.	15%
Functioning scores improved by 0.5 points more than placebo (on a scale from 0 to 10).	1.2 points better	vs.	0.7 points better
**What were ABILIFY'S side effects?**			
**Serious side effects**			
21% more people developed akathisia - severe restlessness that makes it hard to keep still	25%	vs.	4%
3% more people developed movement disorders –like Parkinson's disease	8%	vs.	5%
**Symptom side effects**			
6% more people had insomnia	8%	vs.	2%
5% more had blurred vision	6%	vs.	1%
4% more had substantial weight gain	5%	vs.	1%
4% more had fatigue	8%	vs.	4%
3% more had constipation	5%	vs.	2%

WARNINGS ABOUT UNCOMMON LIFE-THREATENING AND VERY SERIOUS SIDE EFFECTS

Young adults using anti-depressants for major depression have a higher risk of suicidal thinking and behavior.
Elderly patients with dementia-related psychosis should not use antipsychotic drugs – **like ABILIFY**–because they increase death.

Antipsychotic drugs cause: Neuroleptic Malignant Syndrome (very high fever and blood pressure, delirium), Tardive Dyskinesia (uncontrollable facial / body movements), Dangerous Heart Rhythms, Seizures, Low White Blood Cells, Trouble Swallowing, Aspiration Pneumonia, Diabetes, Low Blood Pressure, Trouble Regulating Body Temperature
