# Supplementary material for: Developing principles for sharing information about potential trial intervention benefits and harms with patients: report of a modified Delphi survey
Source: Trials. 2022 Oct 8;23:863. doi: 10.1186/s13063-022-06780-1 (PMC9548137; doi:10.1186/s13063-022-06780-1)
Supplement: Supplementary file 5 — Additional file 5. Full round 2 results. [file 13063_2022_6780_MOESM5_ESM.pdf]

File name: Additional file 5

File format: .doc

Title: Full Round 2 Results Table

Description: Full results from round 2 of the Delphi survey

**Question No.**	**Statement**	**Score 1-3**	**% of N**	**Score 4-6**	**% of N**	**Score 7-9**	**% of N**	**Decision**
1 (round one)<br>1 (round two)	Potential harms that are not very serious do not need to be emphasized.	**25**	**14.12**	**47**	**26.55**	**105**	**59.32**	**No consensus**
	Public, Patient and their advocate	11	28.95	8	21.05	19	50.01	No consensus
	Ethics committee member etc.	11	34.39	5	15.63	16	50.01	No consensus
	Industry (inc. medico-legal expert)	1	7.69	4	30.76	8	61.54	No consensus
	Applied researcher	1	6.25	3	18.75	12	75	Consensus
	Clinical trial professionals	3	4.23	21	29.57	47	66.2	No consensus
	Other	0	0	5	35.72	9	64.29	No consensus
4 (round one)<br>2 (round two)	It is okay to use 'positive framing' when describing how severe harms can be.	**88**	**48.89**	**43**	**23.89**	**27.23**	**49**	**No consensus**
	Public, Patient and their advocate	23	57.5	5	12.5	12	30	No consensus
	Ethics committee member etc.	15	46.88	5	15.63	12	37.51	No consensus
	Industry (inc. medico-legal expert)	3	21.43	6	42.86	5	35.72	No consensus
	Applied researcher	11	68.75	2	12.50	2	18.75	No consensus
	Clinical trial professionals	33	46.49	25	35.21	13	18.31	No consensus
	Other	7	50	2	14.28	5	35.72	No consensus
9 (round one)	General potential benefits (such as 'the medicine may help you and your cancer') should be described.	**149**	**82.79**	**25**	**13.89**	**5**	**3.34**	**Consensus**

3 (round two)								
	Public, Patient and their advocate	34	85	5	12.5	1	2.5	Consensus
	Ethics committee member etc.	30	93.76	1	3.13	1	3.13	Consensus
	Industry (inc. medico-legal expert)	12	85.72	1	7.14	1	7.14	Consensus
	Applied researcher	13	81.25	2	12.5	1	6.25	Consensus
	Clinical trial professionals	59	83.1	12	16.91	0	0	Consensus
	Other	8	57.15	4	28.56	2	14.28	No consensus
11 (round one)<br>4 (round 2)	Only the most important potential benefits should be described. If too many are included the reader might become confused. A complete list can be contained in an appendix or online.	**122**	**67.39**	**44**	**24.31**	**15**	**8.29**	**No consensus**
	Public, Patient and their advocate	25	60.98	9	21.96	7	17.08	No consensus
	Ethics committee member etc.	26	81.25	4	12.5	2	6.25	Consensus
	Industry (inc. medico-legal expert)	11	78.58	3	21.43	0	0	Consensus
	Applied researcher	9	56.25	6	37.50	1	6.25	No consensus
	Clinical trial professionals	47	66.21	19	26.76	5	7.04	No consensus
	Other	10	71.43	4	28.56	0	0	Consensus
13 (round one)<br>5 (round two)	Potential harms should be described more fully than potential trial benefits.	**31**	**17.81**	**45**	**25.86**	**98**	**56.31**	**No consensus**
	Public, Patient and their advocate	10	25.64	9	23.07	20	51.28	No consensus
	Ethics committee member etc.	6	18.76	10	31.26	16	50	No consensus
	Industry (inc. medico-legal expert)	6	46.16	2	15.38	5	38.46	No consensus
	Applied researcher	1	6.67	3	20.01	11	73.34	Consensus
	Clinical trial professionals	6	8.7	20	28.98	43	62.32	No consensus
	Other	4	33.34	1	8.33	7	58.33	No consensus

14 (round one)<br>6 (round two)	Only the most common possible harms should be mentioned. This will focus the reader's attention and minimize overload.	**17**	**9.54**	**33**	**18.53**	**128**	**71.91**	**Consensus**
	Public, Patient and their advocate	7	17.08	6	14.64	28	68.29	No consensus
	Ethics committee member etc.	6	18.76	4	12.51	22	68.76	No consensus
	Industry (inc. medico-legal expert)	0	0	0	0	13	100	Consensus
	Applied researcher	1	6.25	5	31.25	10	62.50	No consensus
	Clinical trial professionals	5	7.04	17	23.94	49	69.02	No consensus
	Other	0	0	2	16.66	10	83.34	Consensus
19 (round one)<br>7 (round two)	It's okay to use 'positive framing'. That is, it is okay to say 'this treatment is safe for 90% of the people who take it' instead of 'this treatment causes side effects for 10% of the people who take it'.	**101**	**57.39**	**35**	**19.88**	**40**	**22.73**	**No consensus**
	Public, Patient and their advocate	22	55	6	15	12	30	No consensus
	Ethics committee member etc.	19	59.38	5	15.63	8	25.01	No consensus
	Industry (inc. medico-legal expert)	4	30.76	3	23.07	6	46.15	No consensus
	Applied researcher	11	73.33	1	6.67	3	20	Consensus
	Clinical trial professionals	43	61.42	17	24.29	10	14.28	No consensus
	Other	7	58.33	3	25	2	16.67	No consensus
20 (round one)<br>8 (round two)	Potential harms should be described in pictures as well as words.	**48**	**29.22**	**97**	**54.49**	**29**	**16.29**	**No consensus**
	Public, Patient and their advocate	15	36.59	19	46.35	7	17.08	No consensus
	Ethics committee member etc.	6	18.75	19	59.39	7	21.88	No consensus
	Industry (inc. medico-legal expert)	3	23.07	8	61.52	2	15.38	No consensus
	Applied researcher	5	31.25	9	56.25	2	12.50	No consensus
	Clinical trial professionals	19	26.76	38	53.52	14	19.72	No consensus
	Other	4	33.34	8	66.67	0	0	No consensus

22 (round one)<br>9 (round two)	Potential benefits should be described after harms.	**12**	**6.82**	**91**	**51.71**	**73**	**41.47**	**No consensus**
	Public, Patient and their advocate	5	12.2	15	36.59	21	51.22	No consensus
	Ethics committee member etc.	3	9.39	17	53.14	12	37.50	No consensus
	Industry (inc. medico-legal expert)	0	0	8	61.53	5	38.46	No consensus
	Applied researcher	0	0	8	50	8	50	No consensus
	Clinical trial professionals	3	4.35	37	53.61	29	42.03	No consensus
	Other	1	8.33	9	75	2	16.66	Consensus
23 (round one)<br>10 (round two)	Potential benefits and harms should be beside each other (for example in two columns).	**102**	**57.62**	**61**	**34.46**	**14**	**7.9**	**No consensus**
	Public, Patient and their advocate	25	59.52	13	30.94	4	9.52	No consensus
	Ethics committee member etc.	15	46.89	12	37.51	5	15.63	No consensus
	Industry (inc. medico-legal expert)	4	30.76	6	46.15	3	32.07	No consensus
	Applied researcher	10	57.50	4	25	2	12.5	No consensus
	Clinical trial professionals	43	62.32	23	33.33	3	4.35	No consensus
	Other	8	66.67	4	33.34	0	0	No consensus
24 (round one)<br>11 (round two)	Information about potential benefits or harms should be presented apart by one or more pages.	**9**	**5**	**36**	**20.46**	**131**	**74.44**	**Consensus**
	Public, Patient and their advocate	6	14.28	13	30.95	23	54.76	No consensus
	Ethics committee member etc.	2	6.23	4	12.5	26	81.26	Consensus
	Industry (inc. medico-legal expert)	0	0	4	30.77	9	69.23	No consensus
	Applied researcher	1	6.25	2	12.50	13	81.25	Consensus
	Clinical trial professionals	1	1.47	12	17.64	55	80.89	Consensus
	Other	0	0	1	8.33	11	91.67	Consensus

25 (round one)<br>12 (round two)	Information about potential benefits and harms should be mentioned in more than one place in the leaflet.	**16**	**9.09**	**37**	**21.02**	**123**	**69.82**	**No consensus**
	Public, Patient and their advocate	7	16.66	12	28.57	23	54.76	No consensus
	Ethics committee member etc.	3	9.69	9	29.03	19	61.29	No consensus
	Industry (inc. medico-legal expert)	2	15.38	4	30.76	7	53.83	No consensus
	Applied researcher	0	0	0	0	16	100	Consensus
	Clinical trial professionals	2	2.90	9	13.05	58	84.07	Consensus
	Other	2	16.67	3	25	7	58.33	No consensus
26 (round one)<br>13 (round two)	A complete (detailed) description of the potential harms (and the likelihood of each harm) should be provided in a table in an appendix.	**119**	**67.23**	**47**	**26.55**	**11**	**6.21**	**No consensus**
	Public, Patient and their advocate	28	66.67	11	26.19	3	7.14	No consensus
	Ethics committee member etc.	24	75.01	6	18.75	2	6.26	Consensus
	Industry (inc. medico-legal expert)	6	46.15	5	38.46	2	15.38	No consensus
	Applied researcher	12	75	4	25	0	0	No consensus
	Clinical trial professionals	44	63.77	21	30.43	4	5.8	No consensus
	Other	11	91.66	1	8.33	0	0	Consensus
27 (round one)<br>14 (round two)	Drug fact boxes (see below) divide harms into serious and non-serious. This way of presenting harms is helpful.	**121**	**68.36**	**38**	**21.46**	**18**	**10.16**	**No consensus**
	Public, Patient and their advocate	31	73.81	7	16.66	4	9.52	Consensus
	Ethics committee member etc.	22	68.76	9	28.13	1	3.13	No consensus
	Industry (inc. medico-legal expert)	7	53.84	4	30.77	2	15.38	No consensus
	Applied researcher	14	87.50	1	6.25	1	6.25	Consensus
	Clinical trial professionals	33	62.32	15	21.74	11	15.95	No consensus
	Other	10	83.33	1	8.33	1	8.33	Consensus
