# Supplementary material for: Developing principles for sharing information about potential trial intervention benefits and harms with patients: report of a modified Delphi survey
Source: Trials. 2022 Oct 8;23:863. doi: 10.1186/s13063-022-06780-1 (PMC9548137; doi:10.1186/s13063-022-06780-1)
Supplement: Supplementary file 6 — Additional file 6. Consensus meeting statements. [file 13063_2022_6780_MOESM6_ESM.pdf]

File name: Additional file 6

File format: .doc

Title: Statements discussed at the consensus meeting

Description: List of statements that were discussed at the consensus meeting

**Statements discussed at the consensus meeting (consensus has not been reached following rounds 1 and 2)**	
**Question No.**	**Statement**
1	Potential harms that are not very serious do not need to be emphasized.
4	It is okay to use ‘positive framing’ when describing how severe harms can be.
11	Only the most important potential benefits should be described. If too many are included the reader might become confused. A complete list can be contained in an appendix or online.
13	Potential harms should be described more fully than potential trial benefits.
19	It’s okay to use ‘positive framing’. That is, it is okay to say ‘this treatment is safe for 90% of the people who take it’ instead of ‘this treatment causes side effects for 10% of the people who take it’.
20	Potential harms should be described in pictures as well as words.
22	Potential benefits should be described after harms.
23	Potential benefits and harms should be beside each other (for example in two columns).
25	Information about potential benefits and harms should be mentioned in more than one place in the leaflet.
26	A complete (detailed) description of the potential harms (and the likelihood of each harm) should be provided in a table in an appendix.
27	Drug fact boxes divide harms into serious and non-serious. This way of presenting harms is helpful.
