# Supplementary material for: Developing principles for sharing information about potential trial intervention benefits and harms with patients: report of a modified Delphi survey
Source: Trials. 2022 Oct 8;23:863. doi: 10.1186/s13063-022-06780-1 (PMC9548137; doi:10.1186/s13063-022-06780-1)
Supplement: Supplementary file 7 — Additional file 7. Round 1 & 2 free-text comments. [file 13063_2022_6780_MOESM7_ESM.pdf]

File name: Additional file 7

File format: .doc

Title: Round 1 and 2 free text comment summary

Description: A summary of the comments that were provided by participants following both round 1 and 2 of the Delphi survey.

**Round 1**	**Free text comments were summarised by the study team and summary was presented to round 2 participants**
	• Participants said it was important to clearly describe risks in terms of both how often the side effects arose and how serious they were<br>• Leaflets should avoid using 'you' or 'your child' and use more generic 'some participants' or 'some children'.<br>• Comparison to known medications can be useful when describing potential harms. For example, saying that the probability of a clot following a vaccine is the same as the probability of a clot linked to smoking or pregnancy.<br>• Uncertainty needs to be conveyed (we are usually not sure who will experience a harm).<br>• Ways of presenting benefits and harms information will depend on disease area; participant characteristics; clinical trial phase; and risk and intervention complexity)<br>• Participants stated that it was important not to over-emphasize trial benefits as these often are unknown.<br>• The Drug fact box prompted a number of comments with some participants viewing it as clear, concise and useful as a text breaker and others as confusing and too complicated.
**Round 2**	**Free text comments were summarised by the study team and summary was presented to the consensus meeting participants**
	• Participants mentioned the importance of a balance between severity and frequency (a very rare but very severe side effect should always be listed regardless of frequency).<br>• Patient and public input should be mandatory when preparing participant facing trial documentation describing benefits and harms<br>• Presented information should take into account the wide range of possible audiences where simple language is complemented with more complex information for example in an appendix or via a weblink (but full and balanced information and clear options for participants should always be offered)<br>• It should always be acknowledged that any risk and benefit information is based on existing knowledge and does not necessarily encompass all potential side effects<br>• The reversibility of side-effect is important and should be described<br>• The use of infographics (as opposed to pictures) might be useful for some but not all participant groups
