# Supplementary material for: Developing principles for sharing information about potential trial intervention benefits and harms with patients: report of a modified Delphi survey
Source: Trials. 2022 Oct 8;23:863. doi: 10.1186/s13063-022-06780-1 (PMC9548137; doi:10.1186/s13063-022-06780-1)
Supplement: Supplementary file 8 — Additional file 8. Round 1 free-text comments. [file 13063_2022_6780_MOESM8_ESM.pdf]

File name: Additional file 8

File format: .doc

Title: Full list of round 1 free text comments

Description: Raw data from all participants comments from round 1 of the Delphi survey

I think all the percentages would confuse a lot of patients, and risk mis-understandings
I think researchers should try to be honest. I know that is difficult and judgmental - and it makes it difficult to it is also difficult to generalise. It also has something to do with the integrity of the search, the researcher and the study. Information also needs to have regard to the audience and their background and their ability to understand.
This section is to confusing for non-clinicians
I think this is too complicated
the survey focuses on emphasising info, but I think there is a difference between emphasising (which isn't always necessary) & explaining (which probably is). Also, the survey talks about describing benefits & harms; but I think it isn't always about just describing, it's often about the way things are described
I find some of the questions difficult to understand. Not lay enough.
Many of us lay people have a poor understanding of statistics. I would like stats and percentages to be presented in more than one way
The definitions of serious are regulatory and not intuitively understood by patients
I like this idea... very clear and correct
a bit too detailed for a layman's intelligence you are aiming for with this leaflet
Unsure if the patient would understand this layout about the drug facts
There's a lot of information here which may not be easily understood presented in this way by a lay person
All needs to be in plain English, or and easy read version.
Important not to overload participant with info, especially listing all possible side effects. Personally, I think excessive use of "you" (or "your child") in info sheets could add to the nocebo effect. ("Your child may not benefit; your child may feel sick" etc seems much more aggressive and threatening than, say, "Some children do not benefit; some children feel sick" but ECs object to changing this form of language. A word count I did on an ICF for parents for a UK trial in a paediatric cancer showed that "your child" appeared >100 times.
confusing comparisons given, more clarification should be given
I've assumed all questions and statements are entirely independent of each other and absolutely literal. questions like the last (and others) are likely to ahve poor validity as the stem does not frame the problem, i.e. what does helpful mean? helpful for an investigator to structure their conversation with a patient? helpful for a patient to retain the information? if you include everything that's helpful in a PIL the PIL would then become unhelpful, industry should probably not define helpfulness and a user centred design approach with iterative feedback to develop the SWAT PILs may be needed.
This seems too complicated for most people to understand
Fully informed consent is paramount for the patient. I have been left disabled following two surgeries where I did not receive the appropriate information regarding potential injuries harm of medical devices.

I think the emphasis of harms/benefits is really important and essentially depends on the seriousness. If a drug has the potential to be life-saving and, based on current evidence, only has less serious potential harms then that should really be emphasised. However, if there are potential benefits but very serious possible harms (e.g. fertility issues) then that is very important and should be emphasised.
1) Great research idea 2) Unless HRA / REC buy into the fact that current PIS are not fit for purpose and only satisfy a narrow legal view we will not move forwards, they seem to be strongly averse to evidence 3) The problem of information overload is indirectly picked up in your survey (PIS are currently far too long) 4) Numbers are probably the wrong tool - the pictorial representations have an evidence base for them 5) Uncertainty needs to be factored in - point estimates give an impression of precision which is often not real.
Quantifying harms if possible is helpful
above table may be better if it just said, likelihood of constipation: 5 vs 3, rather than 3% more constipation. Slightly confusing at a glance
While this has a degree of clarity, a number of people will be very scared by the WARNING at the bottom
Public understanding of "risk" would benefit from advice about where to read more e.g. Spiegelhalter's Norm Chronicles and concept of micromorts, and website on uncertainty.
First part of the fact box (How did Abilify help?) is incredibly complicated! It's a blizzard of numbers & comparisons. Whereas the second part of the fact box (What were Abilify's side effects?) is better but not brilliant. Is it saying 8% of 6% had insomnia on treatment? (Why not say 0.0048%?) Or is it saying 2% more participants had insomnia? The average reading age of a UK citizen is 9 years old!
There is a need to highlight potential harms as people are taking part voluntarily in a trial and usually the risk of not doing so is not established as the trial is to determine IF there is a benefit. The questions here seem to be worded to suggest trials will provide benefits but that is not always the case- often a trial may provide societal benefit but with individual risk and no benefit (e.g. a trial of a vaccine to which the person is not likely to be exposed)
I had a problem with quite a few of the questions. You use qualitative terms such as “not very serious”, “potentially serious”, “very rare”, “likely benefits”, “walk further” without indicating what constitutes very serious, likely, further etc. My decisions will be based by balancing the extent of the hazard and the likelihood of it occurring (ie the components of risk). So if a not very serious harm had a likelihood of 1 in 2, I would regard it differently to the same harm with a likelihood of 1 in 100. My experience of PILs from various PPI work over the last 4+ years is that the key areas where they can fall down are: a) length of document and complexity of language (the average national reading age is said to be 9, and The Sun is said to write for a reading age of 8 and with very short text); and b) the failure to express probabilities in terms understandable to most - https://wintoncentre.maths.cam.ac.uk/ is a good source of advice on this. This is mitigated somewhat by the usual offer to have the document explained by one of the researchers (and for potential participants whose intellectual capabilities suggest a need for pictures, I’d have thought there should be an automatic default to personal explanation, rather than bulking all PILs up with pictures). Happy to be contacted to discuss.
I've not seen a Drug facts box before. I like it and thinks it is very useful. Also there isn't too much text to have to read.
I believe that it is unethical to provide patients with false hope. In the examples given early in the questionnaire, the participants clearly did not understand the potential harms adequately and the research team should have taken more time to explain them properly - I would question if 'informed' consent was actually given. In my experience of reviewing CTIMPS and other trials, there are often no known benefits - which is why the trial is being performed. I believe it is important that participants are made fully aware of these so that they can act accordingly should they experience what might be rare, but very serious side-effects. Stating the benefits could not only give false hope, but could compromise the trial results if a placebo effect is reported (although I appreciate the problems with placebo/nocebo effects). I do actually think the Drug Fact box could

be very useful, but this is too confusing, requiring interpretation of two sets of %. I think if made clearer than they would be more beneficial.
Thank you2
Answers to numerous comments are based on the assumption that there is adequate information known with respect to the weighting one can apply to describe risks/harms/benefits (which depends on the trial type). Maybe standardisation can be thought with respect to trial phase type / risk / complexity. I think portrayal of information (and the answers provided) also assume the cohort is adult and not of highly vulnerable status.
I have ticked "undecided" to some questions because sometimes it depends on context
Not everyone will be able to make sense of a table like this. It would be good to have the same information presented in the text of the leaflet as well as an appendix with a table like this.
It is very difficult to write a PIL as the target audience - patients - is not homogeneous. Patients will have been suffering with their condition for a long time and be au fait with treatments etc...might have already taken part in studies. Others will be new to it and would probably be overwhelmed by the above tables
It truly depends on the type of trial, population, risks and expected benefits and harms. These can be very different in different trials.
The information on adverse effects is not only useful at the consent phase, but when events arise to give context
I feel important factors are severity/magnitude and frequency (for both benefits and harms) - this hasn't come through in all the suggestions here (e.g. there was a scenario about severity then the question was about frequency - or vice versa). Interaction of these is what's important in my opinion
the above would cause dreadful confusion.
I find the Warnings section at the bottom much less explanatory than the parts above. Raises questions in my mind e.g how can anti-depressants give a higher risk of suicidal behaviour? What else can be done for them? Does this mean they shouldn't be prescribed to young adults? What kind of death? etc..... A bit more information about what to do if affected would be welcome.
we know that people find it very difficult/impossible to conceptualise risk as "percentage risk". storytelling is a good way to present a picture of what a level of risk means
Comparisons to other medications can be useful (so long as they are accurate). General public perception of risk is poor so can be assisted by anchoring within known examples or another comfortable narrative. A good example recently has been comparing the clot risk with AZ vaccine with the clot risk of smoking, pregnancy, oral contraceptive, COVID itself etc.
This depends very much on the nature of the trial - not every trial includes either a new medication, or testing more treatment against less. When communicating a more complex study (including non-inferiority designs, for example) there are a great many new concepts to share at the same time. Risk and benefit information competes with explaining randomisation (which can be very unsettling for people), the uncertainties of non-trial diagnosis, prognosis and treatment, and data management explanations which often take up a lot of space in the PIS and can really worry people. The more complexity there is in a PIS, the more likely people with special educational needs or people who are socioeconomically excluded are not to want to take part in a trial: "I'm not very good at this paperwork" is a common comment.
In regards to benefits - I think researchers must be careful not to 'over promise' a potential benefit in a PIS.
On 27. Hard to decide, as part of the text above the table was covered by the start of the table on my screen. On 20. Pictures are useful for children but not adults. On 11. There are never, in my experience, a large number of potential benefits. On cancer risk: There is no good way of describing the potential risk; the comparison with flights across the Atlantic is the best of a bad lot, in my opinion. However, the magnitude of the risk must be made clear in lay friendly language. On the example of the possible gastrointestinal side effects, there would have been less of a problem if these had been described in lay language.

The frequency, severity and reversibility of potential harms are all important and thus far, reversibility has not been mentioned
i think you could make it simpler by dropping the percentages - block capitals are really hard for people with low literacy and low vision to read
Messy, confusing and scary for patients
We all know that how benefits and harms are interpreted depends on the bias of the study/trial therefore I'm not entirely sure that offering a style above would be any less or more disingenuous that not giving the information at all.
These Statistics are misleading.
I have long thought if a trial can give the potential side effects why can't it give the benefits. However my concern would be that drug companies would be marking their own homework and thus could overstate the possible benefits. Could this be overcome by using stats when a drug has been passed in another country because surely this is where the harms are taken from?
not sure you can prescribe the answer for all cases; judgement is needed; hence "undecideds" as an answer
Leaflets should be written in a truthful, meaningfull and understandable way. I am informed the average mental age for literacy of the public is surprisingly very low
I think that some of the answers would be … it depends it is difficult to answer without wanting to qualify the decision
Overly complex in some ways eg the numerical presentation. Overly simplistic in other ways - eg just comparing active drug with placebo
I do think that people should be told why they are participating in the trial.
Glad to see that you are aware of framing effects. However there are other information design issues eg. are people really able to understand % probabilities ?
