# Supplementary material for: Developing principles for sharing information about potential trial intervention benefits and harms with patients: report of a modified Delphi survey
Source: Trials. 2022 Oct 8;23:863. doi: 10.1186/s13063-022-06780-1 (PMC9548137; doi:10.1186/s13063-022-06780-1)
Supplement: Supplementary file 9 — Additional file 9. Round 2 free-text comments. [file 13063_2022_6780_MOESM9_ESM.pdf]

File name: Additional file 9

File format: .doc

Title: Full list of round 2 free text comments

Description: Raw data from all participants comments from round 2 of the Delphi survey

This is a useful project. Some studies may over emphasise benefit so I think it should always be made clear that benefit isn't always a known entity
Interesting!
It is not clear what 'emphasised' means in the questions
Deciding if a harm is serious or not should be done in consultation with the patients who have / will be / might take the drug
A lot of the ways in which information is presented will be very different between disease areas or participant groups. A lot of my 'undecided' responses were reflecting this.
Sorry, missed the first two questions, thought they were examples!
Very interesting the different way of presenting 'facts'...
Q14 confusing - dividing into serious and non-serious is a good thing, but the example given was over complicated and therefore not helpful.
In this last question with the table, I think this might be really difficult to do for most interventions that are being tested. If the data in the table are based on a Phase 1 or small feasibility study, are they helpful? In addition, would people know that a difference of 3 points on a score is clinically meaningful?
Perhaps pie charts should be used as well as written info
It would be helpful to have more than one way of giving statistical information. For instance, in the example you use above say " 1 in 10.... or in other words 90%...."
this particular example of drug fact box is not very easy to understand for a lay person, but the principle is good
My criticisms of the survey and the size of this text box remain unchanged. It is unclear why one should change one's opinion based on the agreement or disagreement of unknown others without any reasons provided.
I think the descriptors on the table in the first column as confusing and not needed. I'd prefer it to just say effect on depression = 9 points vs 6 points
Clear tables are easy to find and break up text. I think the life-threatening side effects should be in the forms of a vertical list, rather than a block of text, and advice on what to do if side effects occur (consult the doctor who prescribed the medication/ stop taking mediciation immediately/ call 999). I agree with the use of positive framing, but important to keep descriptions consistant throughout the PIL - so use percentages throughout (rather than 1 in 10 in one section and 10% in another)
I agree dividing into serious and non-serious harms is helpful but I think the drug facts box is way too complicated an example
I was not sure if we were meant to be influenced by the round 1 scores.
I do like the idea of comparing risk to no/placebo treatment and the drug fact box presented at the end is a good example. However the list of uncommon life threatening and very serious side effects that are presented at the end was quite unnerving as there were no statistical risk quantifiers for readers to contextualise that risk. (When I read it, in my head it sounded like those US adverts for OTC medications that are really positive but then reel off a load of side-effects at the end). Similarly I am also supportive of positive framing providing it does not impact informed consent, e.g. does not produce absolute risk perceptions that are lower than the statistical risk information presented. Also there was a statement about adding pictures to the risk information, I presumed this

was pictures as in figures/icon arrays portraying the statistical risk, rather than pictures of side-effects.
For me it's a balance of how common a side effect is and the severity. If a mild side effect is very rare I don't think it needs to be mentioned but if it's frequent it should be. If it's a very severe side effect, I think it should be mentioned regardless of frequency.
Q14. The table is useful for those who are used to interpreting data but for the general public it could be data overload.
I think there is difficulty when using % - raises questions including 'how many participants involved/assessed etc.
The big point for me is to think of the audience and its diversity. The audience/readership for any PIL will be very varied, in terms of educational level, language ability, capacity to concentrate, grasp of numbers and interpretation of risk, etc etc. One approach will not fit all. Catering for the range of capabilities is, of course, very difficult. But key to attempting this is in my view to adopt a layered style, whereby there is (at least) a brief, simple summary at the beginning, followed by a more comprehensive explanation for those who are interested and can cope with it and, if needs be, additionally an appendix or weblinks.
In the first question about 'positive framing' the example and the statement are about different things. The example is about the use of positive framing in communicating frequency of side effects / harms, not about communicating their severity, which is what the statement asks about. I think that potentially use of positive framing should be used with caution when describing more severe potential harms / side effects (e.g. not making any 'value' judgements on what consititutes a common or uncommon event, but it would still be alright to phrase it as '90% of people do not experience...' or '9 out of 10 people have no...')
I found that whilst thinking about the statements my decision was being swayed by the round 1 percentages. I stopped reading them but they were difficult not to see!
Very comprehensively covered
I think answer options 4 & 6 should not be classed as undecided - it's just how much one agrees. Only 5 should be undecided. By choosing 4 or 6 people have chosen a side of the argument.
I think the info always needs to be offered, but as its own section as an appendix after all initial info given
I think the emphasis on severity of harm is misleading - and your scenario 1 is a good example. Sometimes it is the FREQUENCY or DURATION of side effects/harms, even mild ones, that is what we need to know.
I am not sure that we can dictate what order items are placed in because it is unique to each study. It needs to be done in the most logical way for that study. If I had my way I would make patient and participant innput (PPI) mandatory for all studies
In my view, we should regard previously reported side effects as the tip of an iceberg, rather than a complete or reliable indicator of the drug safety profile. Pharmacovigilance is limited by the fact that participants (and triallists) may not document or report all side effects, whereas benefits are usually based on more easily measurable or objective criteria. Previously observed benefits and harms should therefore not be treated with equal weight. We should take a conservative approach and explain that what has been reported previously does not necessarily encompass all potential side effects.
SWAT re: affects of Round 1 scores on Round 2 scores!
I found q1: Potential harms that are not very serious do not need to be emphasized. I wasn't quite clear what emphasise means in this context- does it mean 'mention' 'mention more than other things' 'have it front and centre of all other info'... for me, it made it difficult to answer without some kind of qualifier of degree of emphasis
The reversibility of side-effects is important and should be described
Interesting and thought provoking.

For some of these examples, I have concerns with how the info was presented, but not necessarily related to the question you asked about it. Like in the diagram above, the dividing up of the symptoms makes sense, but the overall diagram is confusing to me. Or in the example with John and the migraines, it seems a bigger problem that he had to go away and research what he thought the harms meant, and didn't know the benefits, more than that the harms listed were mild.
In considering the use of pictures the patient population could be considered but the use of graphics (not necessarily pictures) help a lot of people. I also wonder if the benefits are placed nearer to signatures, will these be used to influence a signature, but I can see the argument for risks and harms first - The most important thing is balance in the explanations and clear options for the patients/participants
The Abilify table itself is very complex so I wouldn't recommend that way of presenting harms, but dividing them into serious and non-serious is a good idea.
