# Supplementary material for: Developing principles for sharing information about potential trial intervention benefits and harms with patients: report of a modified Delphi survey
Source: Trials. 2022 Oct 8;23:863. doi: 10.1186/s13063-022-06780-1 (PMC9548137; doi:10.1186/s13063-022-06780-1)
Supplement: Supplementary file 10 — Additional file 10. Consensus meeting description. [file 13063_2022_6780_MOESM10_ESM.pdf]

File name: Additional file 10

File format: .doc

Title: Consensus meeting description

Description: Full write up of the consensus group meeting

Consensus meeting

Background

The consensus meeting took place via Zoom and consisted of 12 panel members, the co-applicants and two representatives from each stakeholder group. The objectives of this meeting were to (i) confirm statements where agreement had been reached during the Delphi survey; (ii) gain consensus (whether in or out) on the items for which there was no agreement from the Delphi.

Method

In advance of the meeting participants received a brief summary of the results from each round of the Delphi survey. There were three parts to the meeting. The opening session of the meeting began with the presentation of results from the Delhi where consensus had been reached and asked participants to confirm this. The second element asked panel members to reflect on the items where there was no overall consensus from the Delphi survey. The final part of the meeting invited members to consider the final list of statements and to provide any feedback on how these might be used in practice.

Results

The discussion commenced with confirmation from the panel that they were in agreement with items where consensus had been reached. Each statement where there was no consensus was then presented in turn, along with any free text comments from the survey. Discussion was invited on each item. The following results will outline the main themes raised from each statement before moving on to consider how included PrinciPIL statements might usefully be operationalised.

Statement 1; Potential harms that are not very serious do not need to be emphasised.

The terminology from the statement was raised, with both the language around ‘potential harms’ and ‘emphasised’ discussed in detail. Both terms were considered to be open to misinterpretation by a number of panel members. In addition, a representative from industry highlighted the difference in terminology around risk in comparison to that used within pharmacology. A potential risk within this industry refers to a lack of evidence around any potential harms and is therefore not included in the PIS until evidence confirms otherwise. At the same time, one panel member considered it to be important not to be preoccupied with how words are used in a technical context when the purpose of a PIS is for information to be conveyed in a more general context. The focus should always be the patients and the language they understand.

Overall, the group considered there to be a redundancy with the statement given items that had already reached consensus from the Delphi, in particular that harms should be separated into serious and less serious.

Statement 2; It’s okay to use ‘positive framing’ when describing how severe harms can be.

One expert with a background in researching positive framing stated that the benefits of this approach are currently based on research with healthy volunteers. There is a lack of evidence with the use of positive framing with a clinical population and consequently there are issues with how this translates

to this population. Concerns were also raised around the context within which positive framing is used and that there might be a potential for this approach to be coercive rather than informative. It was agreed that the lack of evidence in the clinical population for the use of positive framing meant this statement could not be included.

Statement 3; Only the most important benefits should be described. If too many are included the reader might become confused. A complete list can be contained in an appendix or online.

One panel member considered this statement to be a “non-issue as there are never too many benefits”. It was agreed that this statement was redundant as PIS should already be listing all potential benefits.

Statement 4; Potential harms should be described more fully than potential trial benefits.

There was little discussion as it was very quickly agreed that this statement should not be included.

Statement 5; It’s okay to use ‘positive framing’. That is, it is okay to say ‘this treatment is safe for 90% of the people who take it’ instead of ‘this treatment causes side effects for 10% of the people who take it’.

It was agreed that no further discussion on ‘positive framing’ was required and that this statement should not be included.

Statement 6; Potential harms should be described in pictures as well as words.

This statement generated a great deal of discussion. One panel member opened the conversation by suggesting that this was important since “pictures are universal in a way that words are not”. However, it was also felt that caution should be exercised when using pictures as they might be “open to interpretation”. The use of images in PIS might also be informed by current research around shared decision making. The form that the pictures took was considered important and that the focus should be on the visual representation of benefits and risk as opposed to pictures per se. In particular, the usefulness of using smiley faces or stick figures to convey risk was deemed to be of value.

Statement 7; Potential benefits should be described after harms.

The difference between the potential benefits of the intervention as opposed to the potential benefits to the individual taking part in the trial was discussed and how these might appear at different points in the PIS. Conceptually it was felt that the rationale for the trial, which would always include any potential benefits, would appear at the start of the PIS. Overall, the group agreed that since there was no firm evidence to specify the order in which benefits and harms are discussed, this statement should be ‘out’.

Statement 8; Potential benefits and harms should be beside each other (for example in two columns).

As potential harms are likely to be longer than potential benefits, the group felt that visually this statement made little sense. In addition, the statement was regarded as redundant given that the concept of balance is already covered in the current Principles.

Statement 9; Information about potential benefits and harms should be mentioned in more than one place in the leaflet.

It was agreed that PIS should be clear and concise and that anything that makes it longer and more complicated should be avoided. Repeating benefits and harms in more than one place would not add any value so the group agreed to leave this statement out.

Statement 10; A complete (detailed) description of the potential harms (and the likelihood of each harm) should be provided in a table in an appendix.

This statement could provide an opportunity for research teams to think about layering the level of information given to potential participants. It was recognised that some participants will want condensed information on harms, others will prefer more detailed information and an appendix could provide this. However, one member of the group queried the inclusion of an appendices since it has a relatively uncontrolled nature with others questioning the length that this might make the PIS. A list of trusted sources to find more information was put forward as offering an alternative to an appendices

Statement 11; Drug fact boxes divide harms into serious and non-serious. This way of presenting harms is helpful.

Whilst drug fact boxes are a useful clinical tool, there is a level of uncertainty around a clinical trial that is not easily transferable into this technique. Consequently, no consensus was reached.

Operationalising the Principles

In the final part to the consensus meeting, panel members were invited to consider ways the current statements might be simplified or combined where appropriate, without losing any of their original meaning.
