# Supplementary material for: Developing principles for sharing information about potential trial intervention benefits and harms with patients: report of a modified Delphi survey
Source: Trials. 2022 Oct 8;23:863. doi: 10.1186/s13063-022-06780-1 (PMC9548137; doi:10.1186/s13063-022-06780-1)
Supplement: Supplementary file 11 — Additional file 11. Final set of principle statements. [file 13063_2022_6780_MOESM11_ESM.pdf]

File name: Additional file 11

File format: .doc

Title: Final set of PrinciPIL statements

Description: Final set of PrinciPILs following the Delphi survey and consensus meeting with explanation

**Consensus in': Over 70% of respondents agreed with the following statements**		**Set of Core Principles as approved by the consensus meeting**	
2	Potentially serious harms need to be emphasized, even if they are very rare.	**1.** (2,18) *	All potential harms of the intervention should be listed. This includes:<br>• common as well as rare potential harms<br>• indirect potential harms (for example to conceiving a child, pregnancy, or breastfeeding)
3	Potential benefits and harms of a clinical trial need to be compared with what happens if the participant does not take part in the trial.	**2.** (15)	The harms should be separated into serious (life threatening, causing permanent damage) and less serious (like a mild headache that goes away quickly).
7	The most likely potential benefits should be described.	**3.** (17)	The fact that not all potential harms are known needs to be explicit. Also, sometimes harms are discovered after the trial begins. As soon as they are discovered*, participants need to be told about them.
8	Any likely benefits to the participant (including embryos, foetus, nursing infants) should be described.	**4.** (7, 8, 9, 10)	All potential benefits of the intervention should be listed. This includes:<br>- General potential benefits (such as 'the medicine may help you and your cancer') should be described.<br>- Concrete, specific potential benefits (such as 'this medicine is designed to enable you to walk farther before becoming breathless') should be described.<br>Likely benefits to the participant (including embryos, foetus, nursing infants) should be described.
10	Concrete, specific potential benefits (such as 'this medicine is designed to enable you to walk farther before becoming breathless') should be described.	**5.** (3)	Potential benefits and harms of a clinical trial need to be compared with what happens if the participant does not take part in the trial.
15	The harms should be separated into	**6.** (20)	Suitable visual representations are recommended where appropriate to describe potential intervention

	serious (life threatening, causing permanent damage) and less serious (like a mild headache that goes away quickly).		benefits and harms, such as the happy and sad faces: 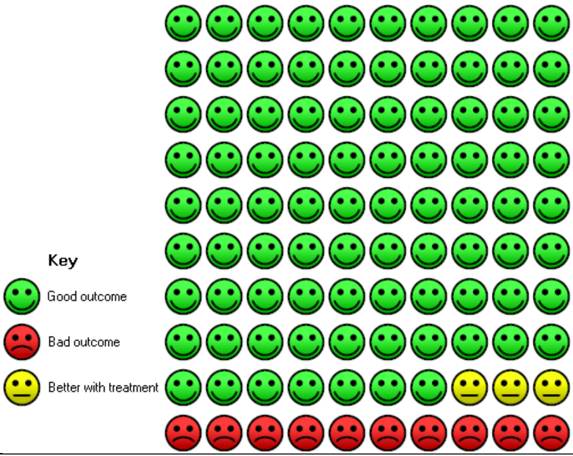
16	Not all potential harms are known, especially for new treatments that have not been studied extensively. Participants need to know that not all potential harms can be listed.	**7.** (Negated 24)	Information about potential benefits and harms should not be presented apart by one or more pages
17	Sometimes harms are discovered after the trial begins. As soon as they are discovered, participants need to be told about them.		
18	Risks to conceiving/fathering a child, pregnancy, or breastfeeding should be emphasized.		
21	Potential trial harms should be described in such a way that they can be compared to what would happen if participant did not take part in the trial.		
Overall, the group proposed that benefits and risks should be grouped together for clarity. Consequently, statements were reordered with items 7,8,9 and 19 relating to benefits grouped together and similarly statements 2 and 18 relating to risk categorised together. The heteronormative language 'fathering' was also removed from statement 18. Statements 3,15 and 17 were considered to be instructive around the communication of risk and benefits and consequently warranted being individual principles. Lastly, the group added the agreed additional item relating to visual representation.			

There was a great deal of discussion around the language used to communicate risk and harm, particularly around the use of instructive terminology. It was suggested that the PIS should only be seen as a supportive tool to aid further communication with the person consenting into a proposed study. Further areas where there is a current lack of evidence was also noted; (i) there is a lack of evidence with the use of positive framing with a clinical population; no firm evidence to specify the order in which benefits, and harms are displayed in the PIL
